# Development and Validation of a Multimodal Optico-Radiological Image System for Neurosurgical Guidance: A Proof of Concept

**DOI:** 10.7759/cureus.81310

**Published:** 2025-03-27

**Authors:** Dilip Bhandari, Mudathir Bakhit, Yuichiro Hayashi, Ryo Hiruta, Kiyoshi Saito, Kensaku Mori, Masazumi Fujii

**Affiliations:** 1 Department of Neurosurgery, Graduate School of Medicine, Fukushima Medical University, Fukushima, JPN; 2 Department of Neurosurgery, Fukushima Medical University, Fukushima, JPN; 3 Department of Intelligent Systems, Graduate School of Informatics, Nagoya University, Nagoya, JPN; 4 Department of Neurosurgery, Fukushima Rōsai Hospital, Iwaki, JPN

**Keywords:** 3d surgical navigation, brain tumors, ct scan, glioma, intraoperative mri, mri, multimodal neuroimaging, neuroimage, surgical microscope, surgical navigation system

## Abstract

Introduction

Advancements in neurosurgery have integrated imaging modalities like fluorescence imaging and neuronavigation to enhance tumor resection and functional preservation. However, aligning intra-operative optical data, such as 5-aminolaevulinic acid (5-ALA) fluorescence and direct cortical stimulation (DCS) tags, with radiological images remains challenging due to brain shift. To address this, we developed the Multimodal Optico-Radiological Image (MORI) platform, a proof-of-concept system integrating intra-operative optical imaging with MRI/CT for improved surgical visualization.

Methods

We evaluated MORI in 19 brain tumor surgeries near eloquent or deep-seated areas. The system comprised (1) optical image capture, (2) 3D surface reconstruction from stereo optical images, (3) registration of optical and radiological images using the iterative closest point (ICP) algorithm, and (4) visualization. Accuracy was validated by measuring registration errors between anatomical landmarks.

Results

MORI reconstructed 3D brain surfaces, integrating fluorescence and functional mapping with MRI. The system achieved an average registration error of 2.2 mm across 10 cases. Case studies demonstrated precise overlay of DCS tags onto MRI for eloquent area localization and 5-ALA fluorescence for tumor margin delineation. Additionally, MORI converted conventional 2D surgical videos into a 4D surgical record for timeline-based integration.

Conclusion

MORI enhances neurosurgical precision by dynamically integrating optical and radiological imaging. Future advancements, such as automation and surgical microscope integration, could refine it into a robust navigation tool, improving intra-operative decision-making, surgical education, and patient outcomes while advancing neurosurgical research.

## Introduction

Advancements in neurosurgery have led to the integration of various optical diagnostic technologies during surgery, significantly enhancing intra-operative decision-making and patient outcomes. Notable examples include fluorescence imaging techniques, such as 5-aminolaevulinic acid (5-ALA) for high-grade glioma surgeries [1,2] and indocyanine green angiography for vascular bypass procedures [3]. Additionally, cerebral blood flow (CBF) imaging techniques, such as laser speckle monitoring and the fusion of microscopic images with neuroimaging mapping data, are becoming increasingly commonplace in neurosurgical practice [4,5].

Recent technological advancements extend beyond traditional visible-light visualization, offering real-time insights into tissue characteristics and blood flow dynamics. These innovations have improved surgical precision, enhanced the extent of tumor resection in brain tumor surgeries, and contributed to better overall patient outcomes [6]. However, despite these advancements, significant challenges remain in integrating intra-operative optical imaging with pre-operative and intra-operative diagnostic imaging modalities, such as MRI, CT, or PET scans.

For instance, fluorescence imaging with 5-ALA is useful in identifying residual tumor tissue during surgery, but precisely aligning fluorescence regions with pre-operative MRI data is challenging. Similarly, cerebral blood flow imaging captured intra-operatively often cannot be seamlessly overlaid onto radiological images. During functional brain mapping, electrodes are used to stimulate regions of the brain while excising a tumor or lesion [7]. For example, stimulation of eloquent areas can result in speech arrest, indicating critical language regions. Direct cortical stimulation (DCS) label tags or markers are typically placed to denote these regions, serving as references for surgeons during tumor excision. However, accurately associating these labeled regions with radiological images often suffers from misalignment, complicating the integration of functional and radiological data and hindering precise clinical evaluations.

To address these limitations, we developed the Multimodal Optico-Radiological Image (MORI) platform, a proof-of-concept system designed to integrate intra-operative optical imaging with pre-operative and intra-operative radiological datasets, such as MRI, CT, and PET scans. Unlike previous approaches that rely primarily on either 2D overlays or rigid navigation systems, MORI enables 3D reconstruction of the surgical field from optical images and registers them with radiological data, offering a spatially coherent, multimodal view of functional and anatomical information.

This integration establishes a flexible and scalable framework for multimodal surgical guidance. Although the current implementation requires manual image acquisition and offline processing, reflecting the constraints of our hardware setup. It lays the groundwork for future systems capable of real-time integration with surgical navigation platforms. Ultimately, MORI aims to bridge the gap between optical and radiological imaging to improve intra-operative decision-making, enhance functional preservation, and support safer and more effective tumor resections.

## Materials and methods

The research was conducted between 2019 and 2022 at the Neurosurgical Department of Fukushima Medical University in Fukushima, Japan, in collaboration with the Graduate School of Informatics at Nagoya University. The study focused on patients with brain tumors, especially those with deep-seated tumors, tumors located near eloquent areas, or cases requiring fluorescence-guided surgery. The Ethics Review Committee at Fukushima Medical University reviewed and approved the research (approval no. 2019-174). Informed consent was obtained from all patients or their guardians.

**Figure 1:**
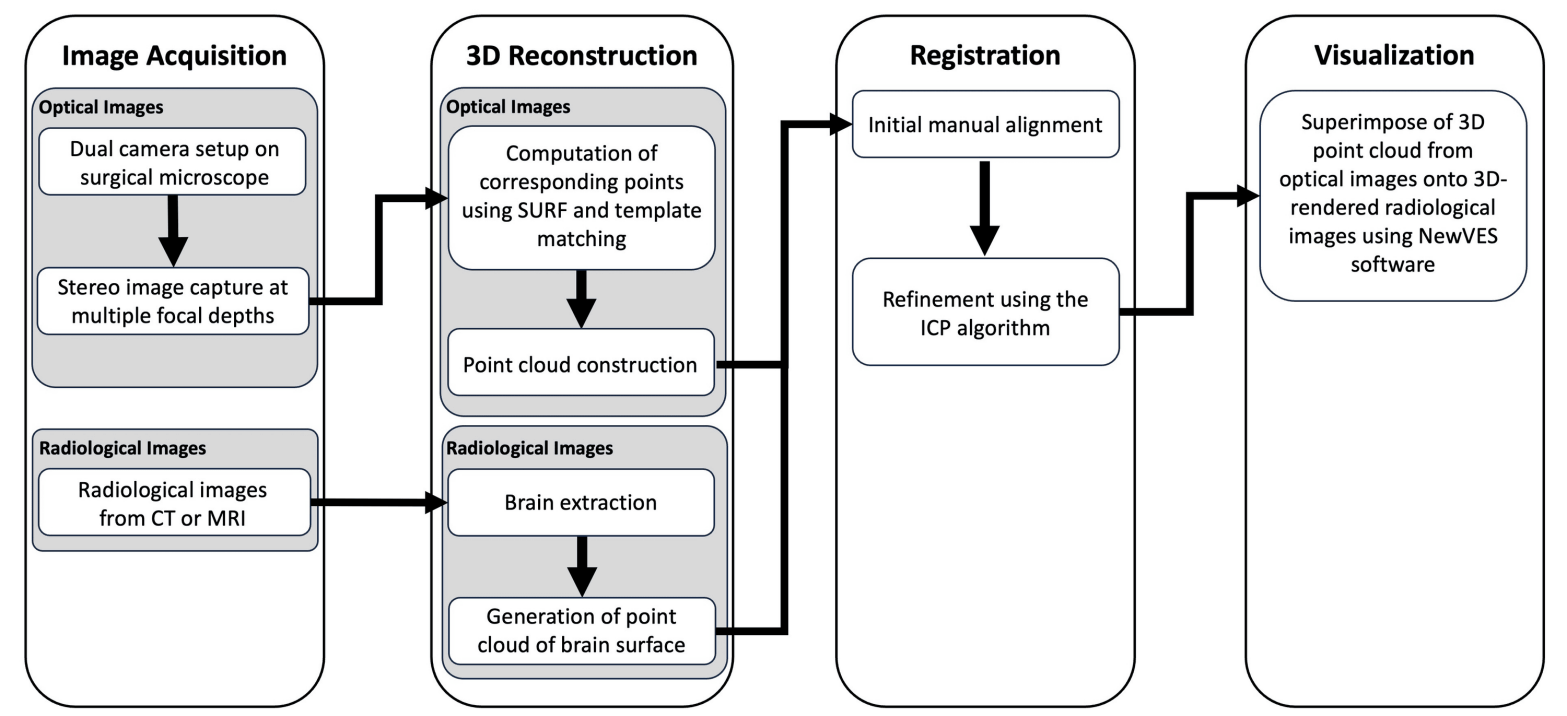
Workflow for generating a Multimodal Optico-Radiological Image (MORI). The process comprises four main steps as follows: image acquisition, 3D reconstruction, registration, and visualization. In the first step, optical images are acquired using a dual-camera setup on a surgical microscope, capturing stereo images at multiple focal depths. Radiological images, such as CT or MRI, are also collected. In the 3D reconstruction step, optical images are processed to compute corresponding points using the Speeded-Up Robust Features (SURF) algorithm and template matching, followed by point cloud construction. Radiological images undergo brain extraction and generation of a point cloud of the brain surface. The registration step involves aligning the 3D point cloud from optical images with the 3D point cloud from the radiological images through initial manual alignment and refinement using the iterative closest point (ICP) algorithm, ensuring the integration of optical and radiological datasets. Finally, in the visualization step, 3D points from optical images are superimposed onto 3D-rendered radiological images and displayed as a Multimodal Optico-Radiological Image (MORI) using NewVES. The image is copyrighted by the author (Masazumi Fujii) of this study (licensed under CC BY-ND 4.0).

**Figure 2:**
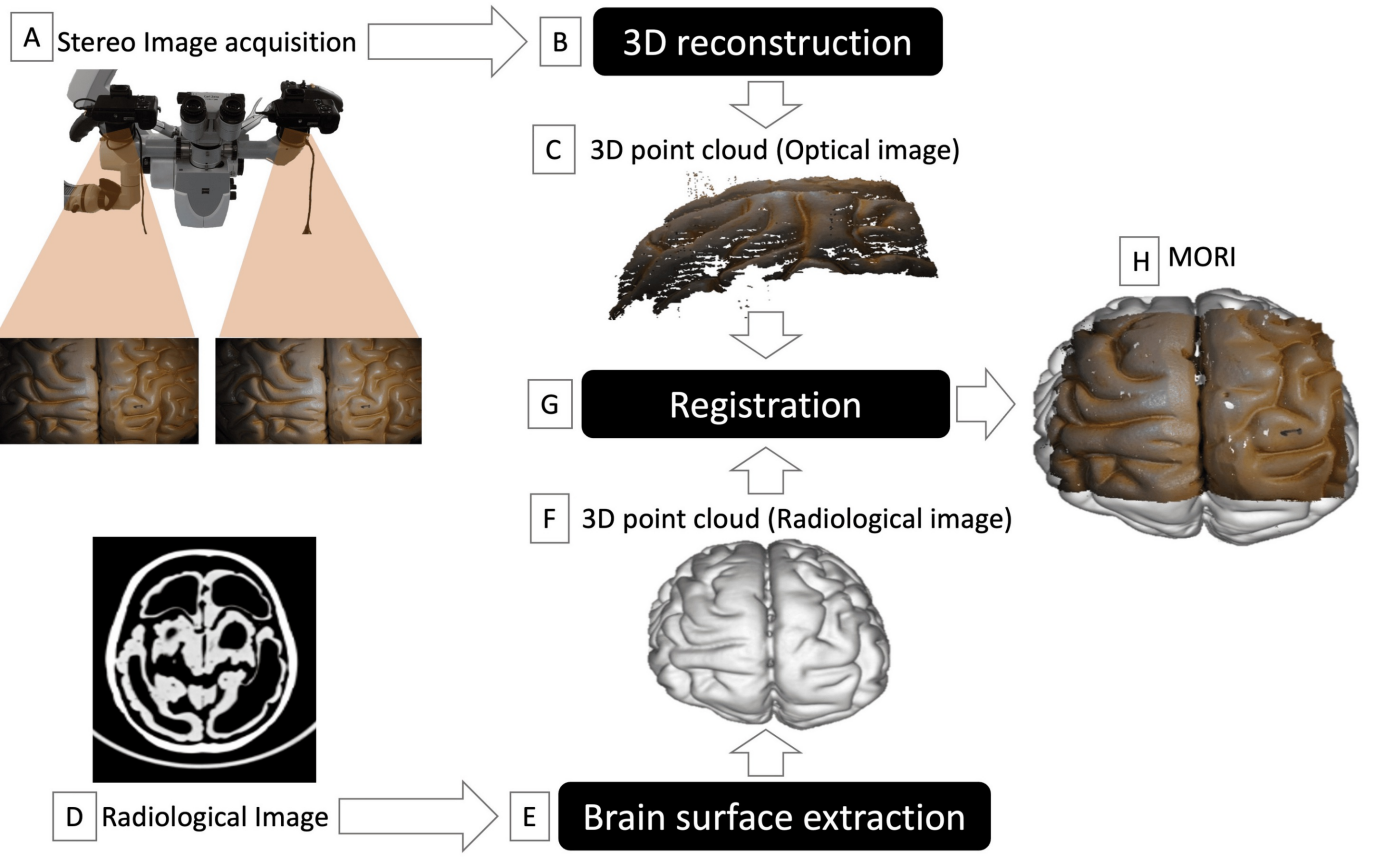
Schematic representation of Multimodal Optico-Radiological Image (MORI) generation using a phantom brain. (A) Stereo microscopic images of the brain surface are captured using two digital cameras, providing detailed 2D surface imagery. (B and C) 3D reconstruction is performed based on the corresponding points computed using the Speeded-Up Robust Features (SURF) algorithm and template matching, enabling the creation of a 3D point cloud of the phantom brain surface. (D and E) A CT image of a phantom head is acquired, and the brain is segmented using the Brain Extraction Tool (BET). (F) 3D point cloud of brain surface is generated from the segmented brain data, transforming the radiological data into a 3D representation. (G) Registration begins with manual alignment of the 3D point cloud of the optical images with the 3D point cloud from the radiological image, followed by a further refinement using the iterative closest point (ICP) algorithm. (H) The aligned datasets are visualized to create a MORI, integrating optical and radiological modalities for a comprehensive representation. The image is copyrighted by the author (Masazumi Fujii) of this study (licensed under CC BY-ND 4.0).

The method comprises four stages as follows: (1) optical image acquisition, (2) 3D shape reconstruction from optical images, (3) registration of the reconstructed 3D shape with radiological images (e.g., CT, MRI), and (4) visualization of both optical and radiological images. Figures 1, 2A-2E provide an overview of the method.

Optical image acquisition

**Figure 3:**
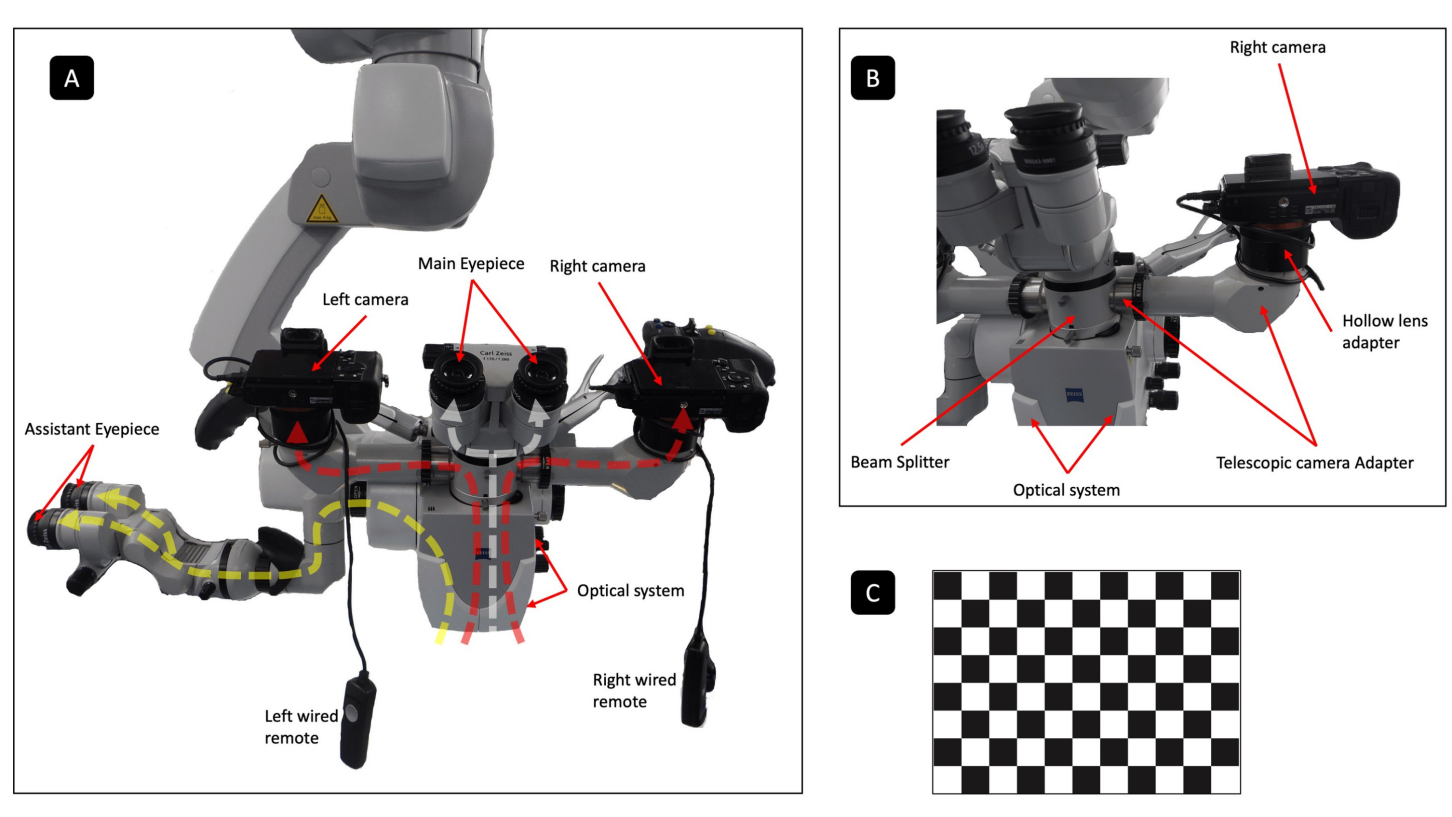
Stereo optical image capturing hardware setup. (A) Two Sony Alpha 7 digital cameras (Tokyo, Japan: Sony Corporation) are mounted on a Zeiss Pentero 900 surgical microscope (Jena, Germany: Carl Zeiss Meditec AG). The optical system splits the operational field image between the main binocular for the surgeon (white dashed line), the assistant binocular (yellow dashed line), and the custom-mounted cameras (red dashed lines). Images are captured through the cameras using wired remote controls. (B) The custom connection setup includes each camera being mounted onto a custom-designed telescopic tube adapter via a hollow lens-style adapter. These adapters connect to opposite sides of a beam splitter, which is connected to the top of the microscope's optical system. (C) A chessboard pattern is photographed using the assembled setup for calibration purposes. The image is copyrighted by the author (Masazumi Fujii) of this study (licensed under CC BY-ND 4.0).

Two Sony Alpha 7 digital cameras (Tokyo, Japan: Sony Corporation) were mounted on a Zeiss Pentero 900 surgical microscope (Jena, Germany: Carl Zeiss Meditec AG) (Figure 3A). The cameras were attached using hollow lens-style adapters, which were connected to custom-designed telescopic tube adapters (Figure 3B). These adapters linked the cameras to the microscope's optical system via a custom-built beam splitter. Both cameras were configured identically for each surgical procedure to ensure uniform image quality and calibration. Images were captured at two focus settings, 350 mm and 500 mm, to accommodate varying depths within the surgical field. Before each procedure, the microscope underwent automatic balancing to maintain optimal alignment and stability during image acquisition. The cameras were controlled remotely using a long-wire trigger, enabling the surgeon to capture images seamlessly without disrupting workflow.

The cameras were aligned to capture slightly different perspectives of the surgical field, simulating binocular vision (Figure 2A). This configuration enabled the simultaneous acquisition of stereo image pairs, which provided the spatial information necessary for performing 3D reconstructions. These stereo images were later processed to create radiological overlays, brain surface models, and functional mapping visualizations.

Images were captured during key surgical stages, including craniotomy, dural incision, tumor site exposure, resection cavity inspection, fluorescence events (e.g., 5-ALA visualization), and functional mapping using direct cortical stimulation. The synchronized capture of right and left images ensured temporal and spatial consistency, which was essential for accurate depth calculation and subsequent integration with pre-operative or intra-operative MRI data.

Before performing surgery, a chessboard pattern was photographed from multiple angles using the mounted cameras for camera calibration, based on Zhang’s method (Figure 3C) [8]. This calibration determined the cameras’ intrinsic parameters (e.g., focal length, optical center, and lens distortion) and the relative position and orientation between the right and left cameras, ensuring accurate geometric measurements. These parameters enable us to obtain relationships among 2D-pixel coordinates in the right and left images and real-world spatial coordinates. Calibration was critical for generating accurate 3D reconstructions, aligning stereo images with pre-operative imaging, and incorporating fluorescence or functional mapping data into surgical workflows. Calibration can also be performed after surgery is complete. Whether calibration is conducted before or after the operation, it is essential to ensure that the camera's attachment to the microscope and its alignment remain consistent.

Captured images were transferred to a computer and organized into separate folders for the right and left cameras. Each image was systematically named to include the following key identifiers: the date, camera side (R for right, L for left), lens focus setting, image type (operative or calibration), and sequence number. This naming convention ensured that each image had a corresponding counterpart, simplifying organization and analysis.

3D shape reconstruction from optical images

The reconstruction of the 3D brain shape began by identifying corresponding points between the stereo image pairs (Figure 2B). These points were mapped into real-world spatial coordinates using the calibrated camera parameters. Initial feature points, representing distinctive surface features such as fluorescent markers or anatomical irregularities, were detected and matched using the Speeded-Up Robust Features (SURF) algorithm, which identified reliable features regardless of variations in size or orientation [9]. To further improve accuracy and enhance the density of corresponding points, template matching was applied using zero-mean normalized cross-correlation (ZNCC), a method robust to brightness variations caused by reflections or lighting inconsistencies [10].

Using the matched points and the calibrated camera parameters, the brain’s 3D shape was reconstructed as a point cloud (Figure 2C). This point cloud, consisting of spatially distributed data points, defined the surface geometry of the brain. The 3D point set refers to these spatially defined data points, forming the foundation for aligning and integrating multimodal imaging data. The resulting 3D representation provided a foundational tool for generating detailed brain surface models, integrating pre-operative imaging, and supporting intra-operative navigation during fluorescence-guided tumor resection or functional mapping procedures.

Registration of the reconstructed 3D shape with radiological images

To integrate optical and radiological datasets, we aligned the reconstructed 3D brain shape from optical imaging with the brain shape extracted from MRI scans. The 3D brain shape derived from optical images was represented as a point cloud, as described in the previous section (Figure 2C). This 3D point set served as the spatially defined data points that formed the basis for aligning the optical and radiological datasets. 

For radiological data, the 3D brain shape was extracted from CT, T1-, or T2-weighted MRI scans using the brain extraction tool (BET) (Figures 2D, 2E). BET is an automated segmentation tool that separates brain tissue from non-brain regions, generating a 3D model suitable for spatial alignment [11]. Next, a 3D point cloud of the extracted brain is created for alignment with the optical data in the subsequent registration step (Figure 2F).

The registration process began with the manual alignment of the optical and radiological 3D brain shapes. This step provided an approximate spatial correspondence by visually matching the two datasets. Following this initial positioning, alignment was refined using the iterative closest point (ICP) algorithm (Figure 2G) [12]. ICP is a mathematical technique commonly used in 3D data analysis that computes a rigid transformation (a combination of translation and rotation) to align two 3D point sets with high precision. The algorithm iteratively minimized the distance between corresponding points in the optical and radiological datasets.

Once the ICP algorithm determined the optimal transformation, it was applied to the 3D brain shape from optical imaging, achieving precise alignment with the MRI data (Figure 2H). This registration process ensured that spatial features, such as tumor boundaries or functional markers, were accurately integrated across optical and radiological datasets. Such alignment was critical for guiding intra-operative decision-making and enhancing the precision of surgical interventions.

Visualization of both optical and radiological images

After the registration process, the aligned optical and radiological datasets were visualized to generate a Multimodal Optico-Radiological Image (MORI) (Figure 2H). This integrated visualization combined the 3D brain shape reconstructed from optical imaging with the radiological data, providing a unified view for analysis and exploration.

The radiological data were visualized using volume rendering (VolR). VolR allowed the neurosurgeon to interact with the MRI data in a dynamic 3D space, enabling detailed visualization of anatomical features. The 3D point sets reconstructed from optical images were overlaid onto the volume-rendered MRI data. During this process, hidden surface removal was applied using the stored depth information in VolR. Hidden surface removal ensured that only visible parts of the optical data were displayed, creating a realistic and accurate integration of optical and radiological information. This step was critical for maintaining spatial coherence and visual clarity in the combined dataset.

For visualization, we used the custom-made application NewVES [13]. NewVES was designed to integrate and view multimodal datasets from multiple viewpoints and directions. It offered precise control over the color and opacity of both the volume-rendered MRI images and the optical 3D point sets, allowing clinicians to focus on regions of interest with enhanced detail. NewVES allows dynamic exploration of critical surgical landmarks, tumor boundaries, or functional areas.

Average distance error measurement

To evaluate the accuracy of the system, we assessed the alignment of optical and radiological datasets by calculating the average distance error between corresponding anatomical points after registration. Five specific landmarks, such as vessel bifurcation points, were selected across a sample of 10 cases. Following alignment using the ICP algorithm, each point in the optical dataset (derived from reconstructed 3D images) was matched to the closest corresponding point in the radiological dataset (from volume-rendered MRI). The spatial position of each point is represented as a set of three values - x, y, and z - which describe its location in three-dimensional space, much like coordinates on a map. The Euclidean distance (di) between each pair of corresponding points was calculated using the formula $di=\sqrt{(X1 - X2)^2 + (Y1 - Y2)^2 + (Z1 - Z2)^2}$, where X1, Y1, Z1 and X2, Y2, Z2 represented the locations of the anatomical landmarks in the radiological data and the 3D point set generated from the optical data, respectively. The total distance for all point pairs was then summed and divided by the number of point pairs to compute the average distance error for each case. This quantitative approach provided an objective measure of alignment accuracy, allowing us to assess the precision and reliability of the system in integrating optical and radiological data for clinical use.

Reproducibility

While this study focuses on demonstrating the feasibility of the MORI platform, we recognize that full real-time implementation and replication may require specific software tools, hardware configurations, and processing pipelines that go beyond the scope of this proof-of-concept. Researchers interested in replicating or building upon this work are encouraged to contact the developers through the Mori Laboratory website at http://www.newves.org/wiki/index.php?English%2FMori%20Laboratory for additional technical details and potential collaboration opportunities.

## Results

In this study, we conducted the experiment during surgeries on 19 patients with brain tumors located in various regions (Table 1). The cohort included eight males and 11 females, with a mean age of 57.4 years. All patients underwent surgeries that incorporated neuronavigation, intra-operative MRI, intra-operative monitoring, and pre-operative administration of 5-ALA as part of the surgical protocol. Among the 19 cases, eight patients underwent awake craniotomy. Performing this experiment during surgery did not influence intra-operative decision-making. Furthermore, no adverse events were observed during the surgeries or in the immediate post-operative period.

**Table 1:** The clinical sample characteristics and the mean distance error. Average (SD) was 2.17 (0.04).

Case number	Age (years)	Sex	Type of tumor	Location of tumor	Awake craniotomy	The mean distance error (mm)
1	36	Female	Glioma	Left middle and inferior frontal	Yes	2.14
2	68	Female	Glioma	Right temporal lobe	No	2.17
3	56	Male	Glioma	Right temporal lobe	No	2.25
4	34	Male	Glioma	Left parietal lobe	Yes	2.19
5	72	Female	Glioma	Right superior frontal	No	2.13
6	61	Female	Glioma	Right insular cortex	No	Not done
7	48	Female	Ependymoma	Third ventricle	No	Not done
8	77	Male	Glioma	Left temporal lobe	Yes	Not done
9	36	Male	Glioma	Left parietal lobe	Yes	2.16
10	52	Female	Glioma	Right insular cortex	No	Not done
11	73	Male	Glioma	Right parietal lobe	No	Not done
12	69	Female	Glioma	Left insular cortex	Yes	2.15
13	44	Male	Glioma	Left temporal lobe	Yes	2.21
14	81	Female	Glioma	Right frontal lobe	No	Not done
15	62	Female	Glioma	Right frontal lobe	No	2.22
16	64	Male	Glioma	Left insular cortex	No	2.12
17	75	Female	Glioma	Right temporal lobe	No	Not done
18	43	Female	Glioma	Left parieto-occipital lobe	Yes	Not done
19	39	Male	Glioma	Left frontal lobe	Yes	Not done

**Figure 4:**
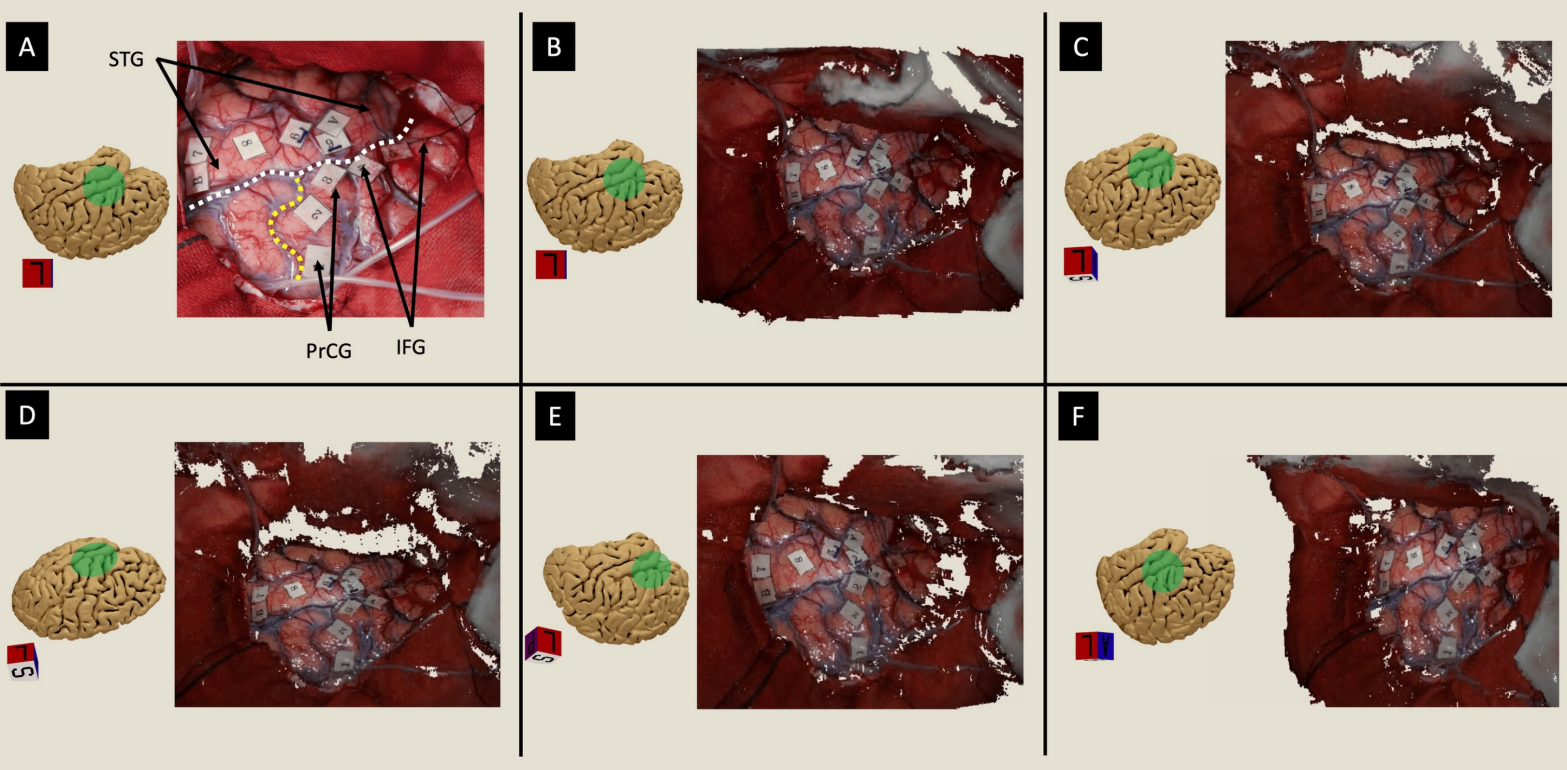
Visualization of a 3D-rendered stereo optical image from different viewpoints. This example is from a patient with a left insular glioma who underwent awake surgery (case number 12 in Table 1). (A) A photograph of the surgical field shows the craniotomy exposing the left inferior frontal gyrus (IFG), superior temporal gyrus (STG), inferior part of the precentral gyrus (PrCG), central sulcus (yellow dashed line), and Sylvian fissure (white dashed line). Brain mapping tags are visible, representing the results of a picture-naming task performed during direct cortical stimulation. Tags labeled "A" and "B" denote the anterior and posterior boundaries of the tumor on radiological images, respectively. For details of the numbered tags (1-9), refer to the subheading "Demonstrative Clinical Application 1" in the Results section. The constructed 3D-rendered optical image is visualized from various viewpoints as follows: the top of the surgical field (B), a superior-tilted viewpoint (C), a more superior-tilted viewpoint (D), a posteriorly tilted viewpoint (E), and an anteriorly tilted viewpoint (F). The orientation box indicates A: anterior, L: left, P: posterior, and S: superior. For a better visualization experience watch Video 1. The image is copyrighted by the author (Masazumi Fujii) of this study (licensed under CC BY-ND 4.0).

The 3D reconstruction of the brain surface was successfully performed using images captured from the surgical microscope during surgery. Figures 4A-4F illustrate the reconstructed 3D shape of the surgical field from five different viewpoints, confirming the successful restoration of the brain surface’s 3D geometry. Notably, the reconstruction encompasses not only the exposed brain surface but also the surrounding surgical field, demonstrating the broader applicability of the method.

The reconstruction was achieved using a single layer of stereo images, which provided a detailed 3D representation of the surgical field. However, as expected, parts of the field not captured within the stereo image layer lacked information, resulting in localized data loss. A demonstration of the 3D reconstruction can be seen in Video 1, which provides a dynamic visualization of the restored surgical field.

**Video 1:** Visualization of a 3D-rendered optical image from different viewpoints. This example is from a patient with a left insular glioma who underwent awake surgery. A stereo-optical image was created from a pair of 2D photos taken by two digital cameras mounted onto a surgical microscope. The surgical field shows the craniotomy exposing the left inferior frontal gyrus, superior temporal gyrus, and Sylvian fissure (white dashed line). Brain mapping tags are visible, representing the results of a picture-naming task performed during direct cortical stimulation. Tags labeled "A" and "B" denote the anterior and posterior boundaries of the tumor on radiological images, respectively. For details of the numbered tags (1-9), refer to the subhead "Demonstrative clinical application 1" in the Results section. The constructed 3D-rendered optical image is visualized from various viewpoints. The video is copyrighted by the author (Masazumi Fujii) of this study (licensed under CC BY-ND 4.0).

Demonstrative clinical application 1

**Figure 5:**
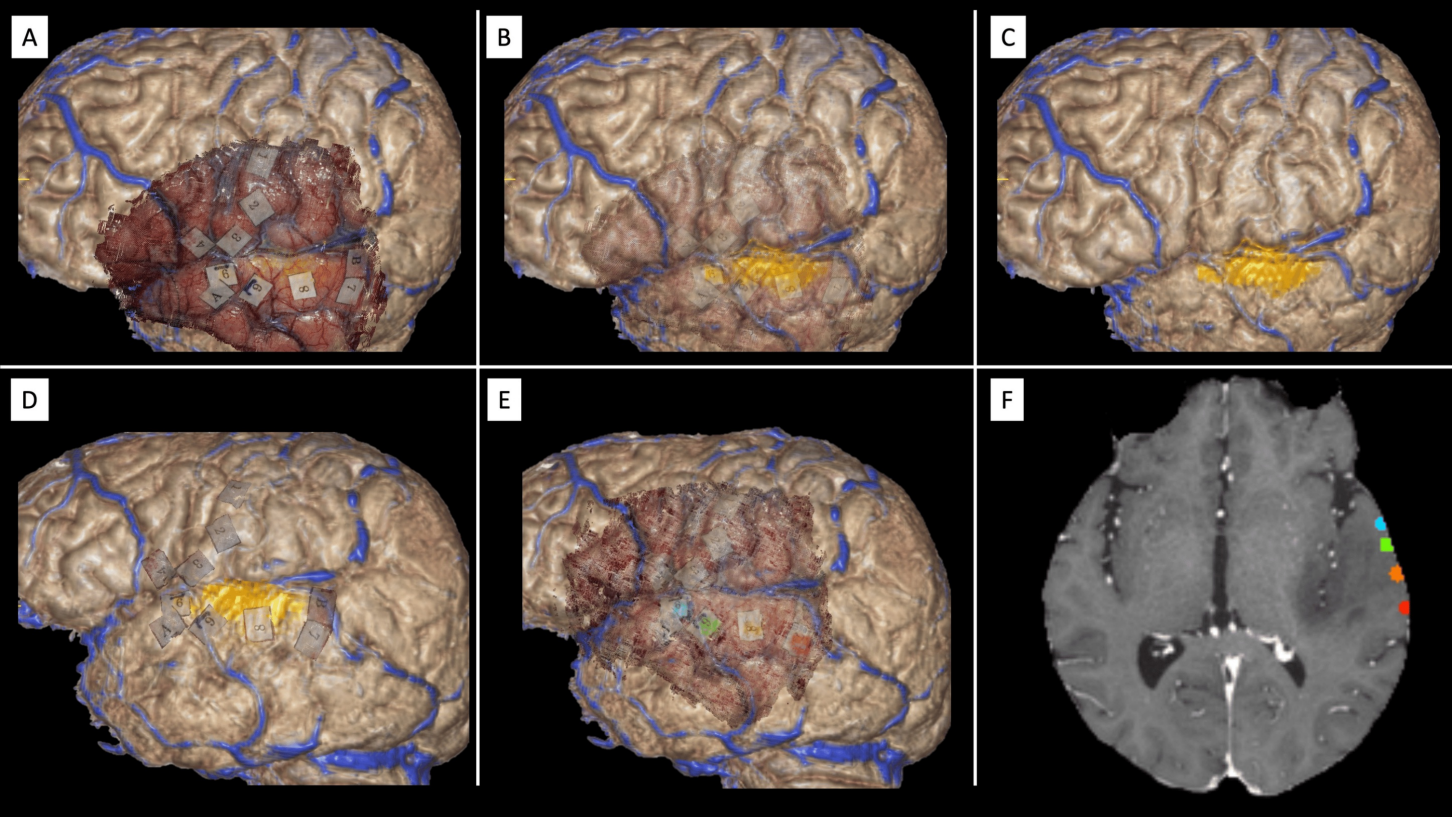
Demonstrative clinical application 1. (A) In the same patient from Figure 4, the Multimodal Optico-Radiological Image (MORI) integrates the surgical field, including cortical map tags, with a 3D-rendered brain extracted from pre-operative MRI. (B and C) Adjusting the opacity of the optical images allows visualization and verification of the alignment accuracy between the optical and radiological modalities. In this case, the venous system alignment between both modalities is confirmed to be accurate. (D and E) Using visualization software (NewVes), selected cortical tags can be color-coded, extracted, and exported for further analysis. (F) The extracted tags are superimposed onto a 2D radiological image for enhanced visualization. In this example, tags 6-9 in the superior temporal gyrus are color-coded as green, red, orange, and blue, respectively. For a better visualization experience watch Video 2. The image is copyrighted by the author (Masazumi Fujii) of this study (licensed under CC BY-ND 4.0).

A 69-year-old female patient with a left-sided insular glioma underwent awake craniotomy for intra-operative cortical mapping of language and motor areas adjacent to the lesion (case number 12 in Table 1). During awake cortical mapping, language and motor areas were identified in close proximity to the tumor and marked with numbered tags. Regarding the mapping shown in Figures 4A-4F, 5A-5F, in response to a four-milliampere cortical stimulation, the patient exhibited the following: label 1 - dysarthria; label 2 - speech arrest; label 3 - semantic paraphasia; and label 4 - speech arrest. No abnormalities were detected at labels 6 through 9. Upon increasing the stimulation to six milliamperes, regions labeled 6-9 expressed pure word deafness.

These functional mapping labels were integrated successfully with the patient’s radiological images (Figures 5A-5F and Video 2). A 3D-rendered optical image of the surgical field, including the mapping tags, was superimposed onto a 3D brain surface generated from the MRI, providing a comprehensive view of the tumor and surrounding anatomical structures (Figure 5A). The tumor could be highlighted in any color (yellow in Figures 5B-5D) to illustrate its spatial relation with the labeled functional areas. The transparency of the optical and radiological images is adjustable, enabling checking the alignment by studying anatomical structures' overlap (e.g., gyri, sulci, and veins) to confirm the registration accuracy between the two imaging modalities. For example, veins visible in the 3D-rendered radiological image were shown to be continuous with those observed in the optical image (Figures 5A-5C). Also, the manipulation of the opacity allows the study of the spatial anatomical relationship between a deep-seated tumor and the superficial DCS label tags, as seen in Figures 5A-5F and Video 2.

**Video 2:** The application of the Multimodal Optico-Radiological Image (MORI) to integrate 3D rendered optical and radiological images. This example is from a patient with a left insular glioma who underwent awake surgery. MORI can display the integration of the optical images of the surgical field, including the brain mapping labeling tags. Adjusting the opacity of the optical images allows visualization and verification of the alignment accuracy between the optical and radiological modalities. In this case, the venous system alignment between both modalities is confirmed to be accurate. (D and E) Using visualization software (NewVes) selected cortical tags can be color-coded, extracted, and exported for further analysis. (F) The extracted tags are superimposed onto a 2D radiological image for enhanced visualization. In this example, tags 6-9 in the superior temporal gyrus are color-coded as green, red, orange, and blue, respectively. The video is copyrighted by the author (Masazumi Fujii) of this study (licensed under CC BY-ND 4.0).

Furthermore, the mapping label tags can be extracted from the optical image, displayed independently, and superimposed onto the 3D-rendered brain and tumor models (Figure 5D). The tags can also be color-coded using the NewVES application (Figure 5E), exported, and superimposed onto a 2D MRI image, allowing clear identification of their precise anatomical locations (Figure 5F).

Demonstrative clinical application 2

**Figure 6:**
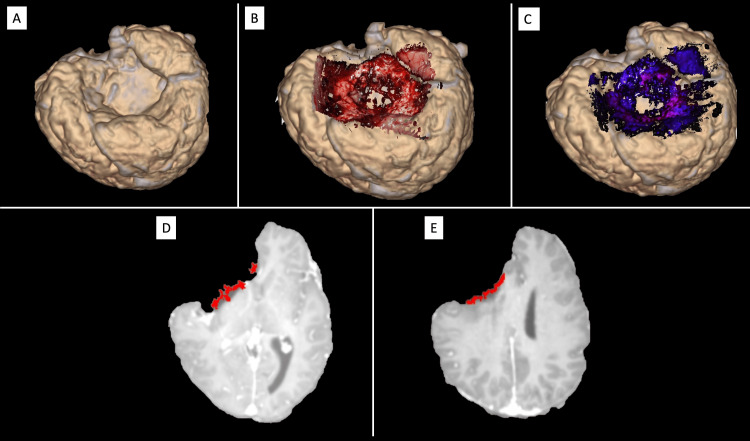
Demonstrative clinical application 2. In this example, we demonstrate how the Multimodal Optico-Radiological Image (MORI) can visualize the superimposition of 5-aminolevulinic acid (5-ALA) fluorescence onto the rendered radiological image in a patient with a right frontal glioma (case 15 from Table 1). (A) A 3D-rendered image of an extracted brain from an intra-operative MRI taken after a later stage of tumor excision, showing the tumor cavity in the right frontal area. (B) The rendered optical image is superimposed onto the 3D brain surface. (C) The optical image of the purple fluorescence emission of 5-ALA is superimposed onto the 3D-rendered radiological surface. Additionally, the area of fluorescence emission can be selected, color-coded, and exported to be superimposed onto a 2D MRI image. (D and E) The fluorescence emission is visualized as a red-colored overlay in the axial plane of the MRI. For a better visualization experience watch Video 3. The image is copyrighted by the author (Masazumi Fujii) of this study (licensed under CC BY-ND 4.0).

In a 62-year-old female patient with a left-sided insular glioma (case number 15 from Table 1), additional imaging and visualization steps were performed to further explore the spatial location of fluorescence observation images of 5-ALA using the MORI system. Figures 6A, 6B illustrate the superimposition of the visible light image onto a 3D brain surface generated from an MRI taken intra-operatively using an MRI T1-weighted contrast-enhanced image following the initial attempt at tumor excision. Subsequently, fluorescence observation images of 5-ALA activation were captured under the same camera conditions and were processed and superimposed on the 3D-rendered brain (Figure 6C). Furthermore, the purple-light-emitting regions of 5-ALA were extracted, red-color coded, and superimposed onto a 2D MRI (Figures 6D, 6E). This multimodal integration confirmed the location of the 5-ALA fluorescence within the MRI dataset, allowing us to identify its proximity to deep brain structures. The MORI system thus provides a comprehensive neuronavigation platform for visualizing critical tumor margins and functional regions in a clinically meaningful way.

Demonstrative clinical application 3

**Figure 7:**
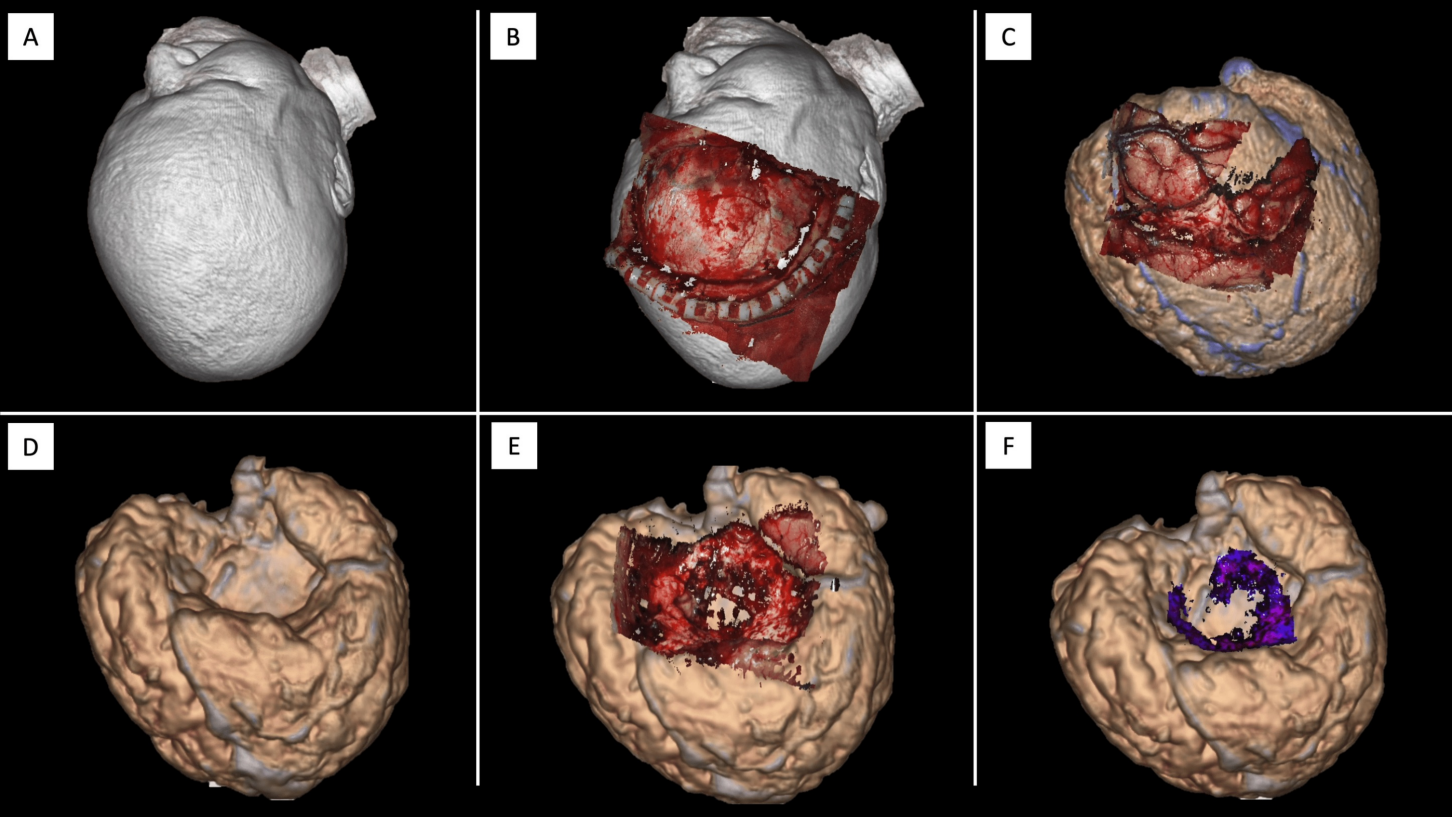
Demonstrative clinical application 3. Using the data from the same patient from Figure 6 (case number 15 in Table 1), we demonstrate how Multimodal Optico-Radiological Image (MORI) can serve as a documentation or registry tool for a surgical timeline by visualizing the integration of rendered optical and radiological images at different time points, creating a 4D image. (A) A 3D-rendered volume of the patient’s head from a CT scan. (B) An optical image taken after the craniotomy and before the dural opening is superimposed onto the rendered scalp. (C) An optical image taken during the early stage of tumor excision is superimposed onto the 3D-rendered brain surface extracted from the pre-operative MRI. (D) A 3D-rendered image of the extracted brain from an intra-operative MRI taken after a later stage of tumor excision, showing the tumor cavity in the right frontal area. (E) A rendered optical image taken during a later stage after extensive tumor excision is superimposed onto the 3D brain surface. (F) An optical image taken during 5-ALA activation shows the emitted purple fluorescence superimposed onto the 3D-rendered radiological surface. For a better visualization experience watch Video 3. The image is copyrighted by the author (Masazumi Fujii) of this study (licensed under CC BY-ND 4.0).

As part of the same case described in the subhead "Demonstrative clinical application 2" (case number 15 from Table 1), optical images were collected along different events in the timeline of surgery and superimposed onto either CT or MRI images, generating a 4D surgical optico-radiological record (Figures 7A-7F and Video 3). This integration transformed the conventional 2D surgical images into a comprehensive surgical record aligned with diagnostic imaging, demonstrating the activities in the surgical field throughout the surgery and the integration of CT, pre-operative MRI, and intra-operative MRI data using the MORI system.

**Video 3:** The application of the Multimodal Optico-Radiological Image (MORI) to document surgical events. This video demonstrates the applicability of the Multimodal Optico-Radiological Image (MORI) as a documentation and registry tool for a surgical timeline by visualizing the integration of rendered optical and radiological images at different time points, effectively creating a 4D image. The rendered optical images of the surgical field were constructed during various phases of the surgery: before dural opening, early tumor excision, and later tumor excision. Each of these images was superimposed onto its corresponding radiological images, whether pre-operative or intra-operative scans. The video is copyrighted by the author (Masazumi Fujii) of this study (licensed under CC BY-ND 4.0).

This example began with the superimposition of the surgical field image captured immediately after craniotomy onto a 3D surface generated from a pre-operative CT scan of the head (Figure 7A). This integration allowed visualization of the skin incision and the craniotomy range, as shown in Figure 7B. Following the dural incision, a 3D optical image of the cortical surface after the initial stage of tumor excision was added to the 3D-rendered brain derived from pre-operative T1-weighted contrast-enhanced MRI (Figure 7C). This integration revealed the surgical operation site and its relationship to surrounding anatomical structures.

As the surgery progressed, an intra-operative MRI scan was acquired. A corresponding 3D-rendered surface was generated (Figure 7D). After resuming the procedure, a new set of optical images was captured, processed, and superimposed onto the 3D surface, providing a real-time view of the surgical field aligned with the intra-operative MRI (Figure 7E). Finally, an optic image of the fluorescence emission of 5-ALA, characterized by strong purple illumination, was taken, processed, and superimposed onto the 3D-rendered brain surface (Figure 7F). The fluorescence region on the MRI delineated tumor infiltration, providing critical spatial information about the tumor margins.

As demonstrated in Video 3, the MORI system successfully integrated surgical images with diagnostic imaging, enabling a comprehensive multimodal record of the surgical timeline. This approach offers significant potential for improving intra-operative visualization and post-surgical analysis.

Average distance error measurement

The accuracy was evaluated by measuring the average discrepancy between corresponding anatomical points in the optical and radiological datasets across 10 cases. The resulting average error was 2.2 mm, indicating a reasonable degree of alignment despite the several manual settings in this experiment (Table 1).

## Discussion

The integration of multimodal imaging techniques in neurosurgery has transformative potential, enabling a comprehensive view of both anatomical and functional data. This study demonstrates a proof of concept for the Multimodal Optico-Radiological Image (MORI) platform, which combines intra-operative optical images with pre-operative and intra-operative radiological images, such as MRI and CT. This integration addresses a longstanding challenge in neurosurgery - the accurate alignment of intra-operative optical data, such as fluorescence and cerebral blood flow imaging, with pre-operative and intra-operative MRI or CT scans. Traditional approaches often struggle with real-time alignment due to timing, modality, and perspective discrepancies. The MORI platform bridges this gap by reconstructing 3D shapes from intra-operative optical images and registering them with radiological images, offering real-time visualization of critical surgical information.

One of the most notable achievements in this study is the successful registration of 3D optical images with radiological images using techniques such as camera calibration, SURF-based point matching, and iterative refinement with ICP [8,9,12]. This approach facilitated the precise overlay of functional data, such as mapping tags, onto MRI, as in Demonstrative clinical application 1 (Figures 5A-5F and Video 2). The ability to visualize eloquent brain areas in relation to diagnostic imaging enhances surgical precision, particularly in complex cases involving tumors near critical regions [14-16].

The MORI platform also demonstrated utility in integrating fluorescence imaging data. In Demonstrative clinical application 2, the superimposition of visible-light and 5-ALA fluorescence images onto intra-operative MRI enabled accurate localization of tumor margins in relation to brain anatomy (Figures 6A-6E and Video 3). Proven to be an important tool, 5-ALA plays a crucial role in maximizing the excision of high-grade glioma [1,17]. The capability of merging the 5-ALA fluorescence with pre-operative or intra-operative images is especially valuable for resection guidance in cases involving deep-seated tumors or lesions near eloquent areas, where precise localization is critical for maximizing tumor removal while preserving neurological function. Such techniques align with broader efforts to improve the visualization of glioblastoma margins, a key determinant of patient survival [18,19].

In Demonstrative clinical application 3, the MORI platform extended its functionality by transforming conventional 2D surgical images into a comprehensive 4D surgical record aligned with diagnostic imaging (Figures 7A-7F and Video 3). This innovative approach enables surgeons to track surgical events and correlate findings such as bleeding, instrument positions, and tumor characteristics with diagnostic images along the surgical timeline. This 4D model not only enhances intra-operative visualization but also serves as an invaluable and comprehensive surgical record for interdisciplinary collaboration, legal documentation, surgical education, and research, enabling a comprehensive understanding of the surgical process along both spatial and temporal dimensions.

The MORI platform offers a unique advantage in addressing a critical limitation associated with pre-operative imaging during neurosurgical procedures. Advanced operating rooms equipped with frameless stereotactic navigation and intra-operative MRI are highly effective for achieving maximal glioma resection. However, following procedures such as decapping, gyrectomy, or CSF drainage, brain shift often renders pre-operative imaging inaccurate, significantly compromising its utility in guiding tumor resection [20].

In such scenarios, MORI can significantly enhance navigation systems by integrating multimodal data captured before and after acquiring new intra-operative imaging. For example, MORI enables the localization of intra-operative findings such as 5-ALA fluorescence regions and DCS tags. This integration allows surgeons to maintain real-time insight into evolving functional maps and surgical anatomy.

By combining MORI’s multimodal outputs with updated intra-operative MRI imaging, surgeons can bridge the gap between biological and functional findings, such as fluorescence and DCS tags, and radiological imaging, enhancing surgical precision in complex cases. This approach not only facilitates maximal safe tumor resection but also ensures the preservation of critical neurological functions by providing an updated and comprehensive navigation framework that reflects intra-operative anatomical changes.

An important feature of the MORI platform is its ability to compensate for brain shift by aligning intra-operative optical data, captured after anatomical deformation, with updated intra-operative MRI. Although the current study does not implement continuous real-time tracking of brain movement, the integration of post-shift optical images with intra-operative radiological data allows for updated visualization of anatomical and functional landmarks. This serves as a practical correction mechanism during key surgical stages, such as after tumor resection, gyrectomy, or cerebrospinal fluid drainage when brain shift is most pronounced. Future developments will focus on increasing the frequency of intra-operative image acquisition and automating alignment updates, which would further enhance MORI’s responsiveness to dynamic intra-operative changes.

Despite the promising results, this study is a proof of concept, and several limitations must be acknowledged. The computational processing and integration were conducted outside the live operating room environment. This approach, while sufficient for demonstrating feasibility, does not account for real-time surgical dynamics, such as patient movement, positional shifts, or the time-sensitive demands of the operating room. Validating the MORI platform in active surgical settings will be essential to confirm its clinical utility and ensure seamless integration into surgical workflows.

Additionally, while the average registration error of 2.2 mm is relatively minor, its clinical impact depends on the context. In many neurosurgical applications, such as tumor resections guided by fluorescence or functional markers, this margin of error is acceptable. However, in ultra-precise functional mapping or surgeries involving deep-seated tumors adjacent to critical white matter tracts, even small misalignments could be significant. Notably, error measurement was conducted on only 10 out of 19 cases, and the sample included a mix of tumor types and locations, which may introduce variability. Future work should focus on improving calibration techniques, stereo image acquisition, and exploring more advanced registration methods, including non-rigid or AI-based algorithms, to enhance accuracy and real-time adaptability.

Moreover, not all 19 cases were used for error measurement (only 10 cases) and the sample included a mix of tumor types and locations, which may introduce variability in the results. Further refinements in calibration processes, stereo image acquisition, and the exploration of advanced algorithms could reduce error margins and enhance accuracy.

Importantly, the current implementation requires manual processing and integration steps conducted outside the operating room, which limits its practicality in a clinical setting. We do not endorse the current prototype for clinical use in its present form. However, we recognize that automating the MORI pipeline, including real-time 3D reconstruction, registration, and visualization, is essential for clinical translation. Future versions should be embedded directly into surgical microscopes or integrated with established navigation platforms to eliminate manual steps, streamline workflow, and enhance intra-operative usability. We view this as a critical next step in making the MORI system more accessible, surgeon-friendly, and suitable for routine neurosurgical procedures.

Lastly, the lack of direct integration with existing navigation systems may limit the platform’s immediate adoption in operating rooms that rely on well-established technologies. However, combining MORI’s capabilities with traditional navigation systems could create a hybrid solution that leverages the strengths of both systems, providing surgeons with dynamic, multimodal visualization tools.

Compared to conventional neuronavigation systems, which rely predominantly on pre-operative imaging and rigid alignment methods, the MORI platform introduces a flexible, multimodal integration approach that incorporates intra-operative optical data. While current commercial systems such as Brainlab or Medtronic offer high precision, they do not typically integrate intra-operative fluorescence or mapping data with volumetric radiology in a dynamic and customizable way. MORI’s design allows for the alignment of functional data (e.g., 5-ALA fluorescence, DCS tags) captured at different stages of surgery, offering a more detailed and context-aware visualization of critical anatomy. Although formal cost analysis is beyond the scope of this study, the use of commercially available hardware and open-source algorithms suggests that a future MORI-based system could offer a cost-effective alternative or complement to high-end commercial platforms. Further quantitative studies are needed to evaluate how this multimodal integration influences intra-operative decision-making and surgical outcomes.

Future directions for the MORI platform should explore its integration with advanced imaging modalities such as functional MRI (fMRI) and diffusion tensor imaging (DTI) for tractography. Incorporating these techniques could significantly enhance the platform’s ability to map eloquent brain regions and critical white matter tracts during surgery. By fusing fMRI data, which provides insights into functional areas like motor and language regions, with DTI-derived tractography, MORI could offer a comprehensive visualization of both cortical and subcortical structures.

Such an approach would allow real-time intra-operative visualization of functional networks and their relationship to the surgical target, addressing critical challenges in surgeries involving deep-seated or eloquent-area tumors. For example, integrating fMRI activations and DTI-derived tracts with 5-ALA fluorescence or cortical mapping tags would provide surgeons with a holistic view of both anatomical and functional data. This could improve the precision of tumor resections while minimizing the risk of damage to essential pathways.

In addition, future developments could focus on automating the integration of these multimodal datasets within the MORI framework, reducing manual processing time, increasing the number of stereo images to avoid void regions, and ensuring seamless real-time updates during surgery.

By incorporating these elements, the MORI platform could evolve into a next-generation neurosurgical tool that not only integrates optical and radiological images but also functional and connectivity data, enabling safer and more precise interventions in complex neurosurgical cases.

Furthermore, the incorporation of artificial intelligence (AI) into the MORI platform presents an exciting opportunity for future development. AI-driven algorithms could automate complex tasks such as 3D reconstruction, feature detection, and multimodal image registration, significantly reducing manual workload and improving processing speed. Although AI was not implemented in the current study, conducted between 2019 and 2022, due to technological and resource limitations, recent advances in medical AI offer promising avenues for enhancing real-time intra-operative performance. Realizing this potential will require interdisciplinary collaboration and access to computational infrastructure, but could ultimately transform MORI into a fully automated, intelligent navigation system optimized for clinical use.

## Conclusions

The MORI platform enhances neurosurgical precision by integrating multimodal imaging in real-time, addressing limitations like brain shift and bridging optical and radiological modalities. While the current implementation requires manual processing, future advancements - such as automation, surgical microscope integration, and incorporation of fMRI and tractography - could refine MORI into a robust navigation tool. This technology holds promise for improving intra-operative decision-making, surgical education, and patient outcomes, while also advancing research in neurosurgery and neuroscience.
